# Neurovascular coupling dysfunction associated with cognitive impairment in presbycusis

**DOI:** 10.1093/braincomms/fcae215

**Published:** 2024-06-26

**Authors:** Chunhua Xing, Jianhua Feng, Jun Yao, Xiao-Min Xu, Yuanqing Wu, Xindao Yin, Richard Salvi, Yu-Chen Chen, Xiangming Fang

**Affiliations:** Department of Radiology, Nanjing First Hospital, Nanjing Medical University, Nanjing 210006, China; Department of Rehabilitation, Nanjing Yuhua Hospital, Yuhua Branch of Nanjing First Hospital, Nanjing 210012, China; Department of Radiology, Nanjing First Hospital, Nanjing Medical University, Nanjing 210006, China; Department of Radiology, Nanjing First Hospital, Nanjing Medical University, Nanjing 210006, China; Department of Otolaryngology, Nanjing First Hospital, Nanjing Medical University, Nanjing 210006, China; Department of Radiology, Nanjing First Hospital, Nanjing Medical University, Nanjing 210006, China; Center for Hearing and Deafness, University at Buffalo, The State University of New York, Buffalo 14215, USA; Department of Radiology, Nanjing First Hospital, Nanjing Medical University, Nanjing 210006, China; Department of Medical Imaging, Wuxi Medical Center, The Affiliated Wuxi People's Hospital of Nanjing Medical University, Wuxi People's Hospital, Nanjing Medical University, Wuxi 214023, China

**Keywords:** presbycusis, neurovascular coupling, cerebral blood flow, blood oxygen level-dependent, cognitive impairment

## Abstract

The neuropathological mechanism underlying presbycusis remains unclear. This study aimed to illustrate the mechanism of neurovascular coupling associated with cognitive impairment in patients with presbycusis. We assessed the coupling of cerebral blood perfusion with spontaneous neuronal activity by calculating the correlation coefficients between cerebral blood flow and blood oxygen level-dependent-derived quantitative maps (amplitude of low-frequency fluctuation, fractional amplitude of low-frequency fluctuation, regional homogeneity, degree centrality). Four neurovascular coupling metrics (cerebral blood flow-amplitude of low-frequency fluctuation, cerebral blood flow-fractional amplitude of low-frequency fluctuation, cerebral blood flow-regional homogeneity and cerebral blood flow-degree centrality) were compared at the global and regional levels between the presbycusis group and the healthy control group, and the intrinsic association between the altered neurovascular coupling metrics and the neuropsychological scale was further analysed in the presbycusis group. At the global level, neurovascular coupling was significantly lower in the presbycusis group than in the control group and partially related to cognitive level. At the regional level, neurovascular biomarkers were significantly elevated in three brain regions and significantly decreased in one brain region, all of which involved the Papez circuit. Regional neurovascular coupling provides more information than global neurovascular coupling, and neurovascular coupling dysfunction within the Papez circuit has been shown to reveal the causes of poor cognitive and emotional responses in age-related hearing loss patients.

## Teaser

This study provides evidence of global and regional neurovascular coupling abnormalities in patients with presbycusis and confirms the association with the Papez circuit, revealing its potential as an early marker of cognitive impairment in presbycusis and contributing to early diagnosis and assessment of the disease.

## Graphical Abstract

**Graphical Abstract:**
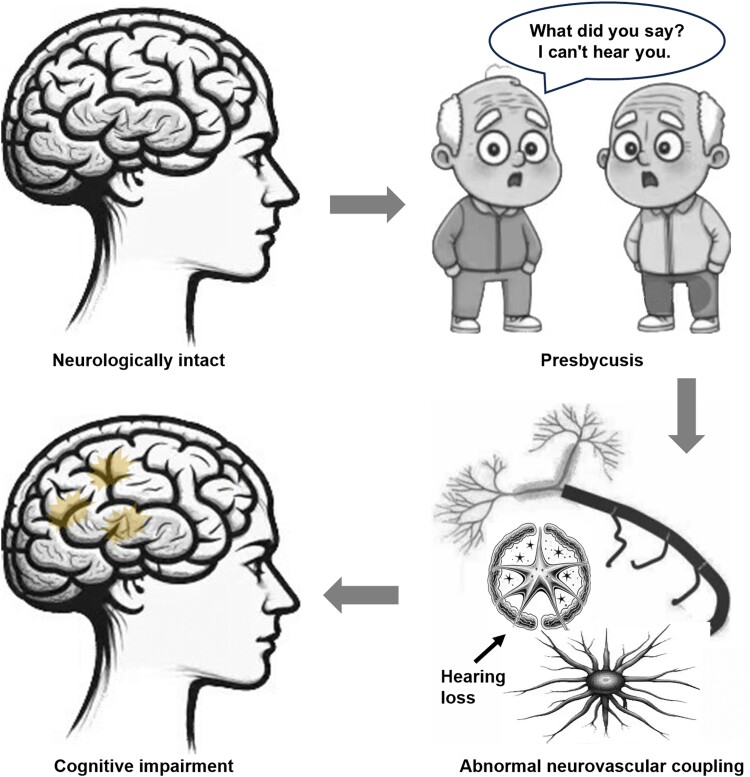

## Introduction

Presbycusis, also known as age-related hearing loss, is the third most common chronic health disorder affecting elderly people.^1^ Typical clinical manifestations first affect the ability to understand high-frequency components of speech and then, with the irreversible loss of cochlear hair cells and auditory nerve damage, gradually progress across frequencies in persons older than 50 years;^2,3^ this disease is a bilateral, multifactorial disease affecting auditory sensitivity. Importantly, numerous studies have shown that age-related hearing loss is independently associated with cognitive impairment and the subsequent development of incident dementia.^4^ Given the large social and economic burden of Alzheimer’s disease (AD) and the fact that hearing loss is a potentially modifiable risk factor for dementia,^5,6^ therapeutic interventions to improve age-related hearing loss are urgently needed. However, a prerequisite for treatment is an understanding of the underlying molecular mechanisms. Therefore, exploring the neuropathological mechanism of cognitive decline in patients with presbycusis and screening for potential biomarkers are highly important.

Advanced multimodal neuroimaging techniques have been established for identifying the neuropathological mechanisms of cognitive disorders in patients with presbycusis. Cerebral blood flow (CBF) and blood oxygen level-dependent (BOLD) signals capture different features in the resting state, revealing the effects of hearing impairment on blood flow and functional changes from the perspective of local blood supply and neuronal activity. Specifically, CBF maps originating from arterial spin labelling (ASL) signals are used to assess cerebral blood perfusion without the use of contrast agents.^7^ Existing studies have shown a downward trend in CBF involving extra-auditory regions^8^ and an increase in CBF in visual-related areas,^9^ suggesting that hearing-impaired patients have reduced auditory attention and some degree of compensation for other senses. Resting-state functional MRI (rs-fMRI) has been widely used to investigate spontaneous brain activity through the detection of multiple aspects of BOLD signals, including the amplitude of low-frequency fluctuation (ALFF), fractional ALFF (fALFF), regional homogeneity (ReHo) and degree centrality (DC). Among these indices, ALFF reflects the spontaneous neuronal activity of each voxel, fALFF (the ratio of low-frequency fluctuation to the entire range) is used to estimate the degree of functional connectivity among voxels, and ReHo measures neuronal synchronization. Previous studies of ALFF and ReHo have reported disruptions in spontaneous neuronal activity in multiple brain regions in patients with presbycusis.^10^ In a study of patients with long-term hearing impairment, fALFF values were significantly elevated in the temporal and insular lobes, confirming that brain regions within different functional networks were involved in brain remodelling.^11^ DC theory explores global brain connectivity, and voxels with high DC values are considered brain hubs. The DC density in the middle frontal gyrus (MFG) was significantly decreased in elderly individuals with hearing loss, which reflects disorders of the brain centre leading to a decrease in higher-order cognitive levels mediated by the frontal lobe.^12^

Recent studies have shown that brain neuronal activity depends on the continuous supply of nutrients such as oxygen and glucose by CBF and on the tight coupling of the formation time and region of the neurovascular unit (NVU) the ‘neurovascular coupling’ (NVC) mechanism which ensures that the regional CBF supply is highly spatially and temporally matched with changes in local brain activity. This ensures a sufficient energy supply to the brain for information transmission and central processing.^13,14^ However, neurovascular decoupling has been observed in patients with schizophrenia, AD and Parkinson’s disease^15-17^ and may be a neuropathological mechanism of brain dysfunction. Positron emission tomography (PET)^18^ measures glucose metabolism, which is one of the most commonly used neurodegenerative biomarkers, and studies have validated ASL CBF as a reasonable and safer substitute for PET, providing noninvasive quantification of CBF. The prominent pathological change associated with age-related hearing loss is a decrease in blood-labyrinth barrier function due to ageing degeneration of the cochlear stria vascularis.^19^ However, the aforementioned studies focused only on a single imaging modality, CBF or rs-fMRI; they ignored the relationship between neuronal activity and vascular injury and did not well reflect the NVC dysfunction manifested by the disjointedness between them, which is likely insufficient to fully elucidate the neurological mechanism of the disease.

Therefore, we explored the cross-modal coupling between brain function and CBF with the aim of elucidating the mechanisms of cognitive impairment in patients with presbycusis from a new perspective. BOLD and ASL perfusion data were collected from patients with presbycusis and healthy controls (HCs). BOLD data were used to calculate ALFF, fALFF, ReHo and DC to reflect neuronal activity, and ASL data were used to measure CBF to represent metabolic needs. First, the across-voxel correlation between CBF and neuronal activity metrics was measured to detect the consistency of the spatial distribution at the whole-brain level (global NVC). Second, we evaluated NVC for a specific region (regional NVC). Finally, the correlation analysis between NVC metrics and cognitive performance was performed.

## Materials and methods

### Participants

#### Study group

Thirty-six patients with hearing loss (21 males and 15 females, age range 51-80 years) from the Otolaryngology Department of Nanjing Hospital Affiliated with Nanjing Medical University were included in this study (presbycusis group). The hearing threshold was measured by pure tone audiometry (PTA). According to the latest grading system in the World Report on Hearing released by the WHO in 2021,^20^ the PTA threshold was computed using four frequencies in the better-hearing ear (the average of 500, 1000, 2000 and 4000 Hz), and the inclusion criterion for hearing loss was that the PTA of the better-hearing ear was ≥20 dB.

#### Control group

Thirty-eight healthy individuals (18 males and 20 females, aged 50-76 years) were recruited from nearby communities and family members of hospital staff and matched for age, sex and education level as the HC group. For HCs, normal hearing was defined as a hearing threshold <20 dB.

All the subjects had normal middle ear function according to a type A tympanometry curve, which was assessed by a Madsen Electronics Zodiac 901 Middle Ear Analyzer (GN Otometrics).

The general exclusion criteria were as follows: an ear condition other than presbycusis affecting the threshold and sensorineural hearing loss, including brain tumour or damage, asymmetry, conductive or hereditary hearing loss, Meniere’s disease, tinnitus^21^ or self-reported hyperacusis;^22^ a history of otolaryngology surgery, noise exposure,^23^ use of otolaryngology drugs or hearing aids; abnormal brain structure; or psychiatric or neurological disorders and other major diseases (i.e. cancer, severe hypertension, diabetes, thyroid dysfunction), as well as any contraindications to MRI (such as claustrophobia and metallic implants). This study was approved by the Medical Ethics Committee of Nanjing Medical University, and each subject provided written informed consent prior to the study.

### Neuropsychological evaluation

All participants underwent a neuropsychological evaluation, with a series of tests assessing cognitive behaviour, executive functioning, language skills and affective symptoms. The digit span test (DST) measures attention and short-term memory; the complex figure test (CFT) and CFT-delay assess visual perception, visuomotor integration and visuospatial recall. The auditory-verbal learning test (AVLT) and AVLT-delay measure episodic verbal learning. The digit symbol substitution test (DSST) measures information processing speed and attention switching. The trail-making test (TMT) is divided into two parts, which are used to assess attention and executive function. The TMT-A measures visuospatial attention and performance speed, while the TMT-B measures mental flexibility and the ability to shift attention. The Montreal Cognitive Assessment (MoCA) is used to assess general cognitive ability. Anxiety and depression were assessed by the Self-rating Anxiety Scale (SAS) and Self-rating Depression Scale (SDS), respectively.

### Data acquisition

All subjects underwent MRI scanning on a 3.0-T MRI system (MAGNETOM Prisma; Siemens Healthcare, Erlangen, Germany) with a 64-channel phased-array head coil. During data acquisition, the subjects were placed in a supine position wearing earplugs and instructed to keep their eyes closed and remain awake without thinking about anything specific. Earplugs and foam padding were used to reduce the influence of noise and involuntary head movements. Functional images were acquired using a gradient-echo planar imaging sequence with the following parameters: repetition time (TR) = 2000 ms, echo time (TE) = 30 ms, flip angle (FA) = 90°, number of time points = 230, slice number = 33, slice thickness = 4 mm without gaps, matrix size = 94 × 94, field of view (FOV) = 220 mm × 220 mm and voxel size = 2.3 × 2.3 × 4.0 mm^3^. Pseudo-continuous ASL based on three-dimensional gradient spin echo (3D GRASE) sequence was used to obtain perfusion images with the following parameters: TR = 4000 ms, TE = 16.86 ms, FA = 180°, slice thickness = 3 mm, gap = 1.5 mm, slice number = 44, FOV = 220 mm × 220 mm, matrix size = 64 × 64, TI1 = 1500 ms, TI2 = 2510 ms and voxel size = 1.7 × 1.7 × 3.0 mm^3^. Structural data were acquired using a 3D T1-weighted magnetization-prepared two rapid gradient-echo sequence with the following parameters: TR/TE = 5000/2.98 ms, thickness = 1 mm, gap = 0.5 mm, slices = 176, FOV = 256 mm × 256 mm, INV1 or TI1 = 700 ms, FA = 4°, and INV2 or TI2 = 2500 ms, FA = 5°, and voxel size = 1.0 × 1.0 × 1.0 mm^3^.

### Blood oxygen level-dependent data processing and functional connectivity metric calculations

BOLD images were preprocessed using Data Processing and Analysis of Brain Imaging (DPABI) toolbox (version 4.5, http://rfmri.org/dpabi) software^24^ based on statistical parametric mapping (SPM 12) implemented in MATLAB R2013b. First, DICOM was converted to NIFTI, the first 10 time points for signal stabilization were removed, and the slice timing and realignment for head motion correction were performed (participants with a maximum head movement over 3.0 mm translation or 3.0° rotation in any direction were excluded; two subjects in the presbycusis group were excluded from the study due to excessive head motion). Then, the images were normalized to the Montreal Neurological Institute (MNI) template with a resampled voxel size of 3 × 3 × 3 mm^3^ by the DARTEL method using the T1 image segment and spatially smoothed with a 6-mm full-width at half-maximum (FWHM) Gaussian kernel (the ReHo and DC values were smoothed in the final step). Detrending was subsequently applied to remove linear trends in the time course. Finally, nuisance covariates, including Friston-24 motion parameters, white matter signals and cerebrospinal fluid signals, were removed via linear regression.

We used DPABI V4.5 software to generate ALFF, fALFF, ReHo and DC maps, where ALFF, fALFF and ReHo were analysed to measure the local spontaneous activity of individual brain regions or voxels.^25^ However, binarized DC was used to measure the significant connectivity between time courses of whole-brain voxels.

For ALFF and fALFF, the time series of each voxel was first transformed into the frequency domain using a fast Fourier transform to obtain the power spectrum. Subsequently, the square root of the power spectrum in the predefined frequency band (0.01-0.08 Hz) was calculated and averaged. Thus, the ALFF value was defined as the average square root within the voxel, and the fALFF value was defined as the ratio of the power spectrum in the given frequency range to the entire frequency band, which contributed to the increased specificity and sensitivity of local spontaneous brain activity detection. The resulting ALFF and fALFF maps were then m-normalized by dividing the value of each voxel by the global mean within the whole-brain mask, and these maps were identified as mALFF and mfALFF, respectively.

For ReHo, Kendall’s coefficient of concordance was used to measure the similarity of the time series of a given voxel to its 26 nearest neighbours within a whole-brain mask. The resulting ReHo map was also m-normalized and defined as mReHo. Next, the mReHo maps were spatially smoothed with a Gaussian kernel of 6 mm FWHM.

Binarized DC was defined as the number of significant connectivities between a voxel and other voxel time courses. The preprocessed BOLD data were first bandpass filtered (0.01-0.08 Hz), and then, the Pearson correlation coefficients between the filtered time courses of each voxel pair and the grey matter mask were calculated, limiting the analysis to correlations above a threshold of *r* = 0.25 to remove the influence of weak functional connectivity due to noise.^26^ Eventually, a 6-mm FWHM Gaussian kernel was used to spatially smooth the binary DC maps. DC density was standardized by m-normalization, which was defined as the mDC parameter.

### Perfusion data processing and CBF calculation

The ASL data processing toolbox ASLtbx (https://www.cfn.upenn.edu/zewang/ASLtbx.php) and SPM8 software (http://www.fil.ion.ucl.ac.uk/spm/) were used to process the ASL data and generate CBF maps by combining ASL difference maps and proton density-weighted reference maps.^27^ The following steps were used: (i) The CBF images were normalized to the MNI standard space by using SPM8 software. (ii) The ASL difference images of healthy subjects were nonlinearly transformed, coregistered with the PET perfusion template in MNI space and subsequently averaged to generate study-specific ASL templates, defined as MNI standard CBF templates. (iii) The individual ASL difference images of all participants were nonlinearly comatched to the MNI standard CBF template. (iv) The non-brain tissue was removed from each coregistered CBF, and a Gaussian smoothed space of 6 × 6 × 6 mm^3^ FWHM was used. (v) The CBF map was standardized by m-division transformation to generate the mCBF parameter.

### Neurovascular coupling quantification

#### Global neurovascular coupling analysis

To enable quantitative evaluation of the global NVC, whole-grey matter (GM) across-voxel correlation analysis was performed between images of neuronal activity (ALFF, fALFF, ReHo and binary DC maps) and cerebral perfusion (CBF maps) for each participant using DPABI software.^28^ Thus, each participant had a correlation value (CBF-ALFF, CBF-fALFF, CBF-ReHo or CBF-DC coefficient) that reflected the consistency of the spatial distribution of neuronal activity and CBF.^29,30^ A two-sample *t*-test was used to compare the correlation coefficients of the four neurovascular modalities between the two groups while controlling for the effects of age, sex and education level.

#### Regional neurovascular coupling analysis

To quantify regional NVC (that is, blood flow or metabolic energy per unit of neuronal activity), we calculated the CBF/ALFF, CBF/fALFF, CBF/ReHo and CBF/DC ratios in a voxel-wise manner for each subject.^31^ A two-sample *t*-test was used and corrected for age, sex and education level to identify significant differences in brain regions between the two groups. Moreover, the mean ratio of each significant cluster was extracted for the subsequent correlation analyses.

### Statistical analysis

#### Clinical variables

The differences in demographic and clinical characteristics and cognitive scale scores between the patients with presbycusis and HCs were statistically assessed with SPSS version 20.0 software. For continuous variables, the independent two-sample *t*-test was used for normally distributed data, and the Mann–Whitney U-test was used for non-normally distributed data. For categorical variables such as sex, the χ^2^ test was used, and *P* < 0.05 was set as the level of statistical significance.

#### Neurovascular coupling metrics

An independent two-sample *t*-test was used to compare the difference in NVC between the presbycusis group and the HCs. For the global coupling correlation coefficients, Bonferroni correction was performed, and *P* < 0.05 was considered to indicate statistical significance. For the regional NVC ratio, multiple comparisons were corrected according to Gaussian random field (GRF) theory, with a voxel *P*-value < 0.001, a cluster *P*-value < 0.05 and two-sided data. Age, sex and years of education were used as covariates.

#### Voxel-wise comparisons

Voxel-wise comparisons were performed to identify the CBF, ALFF, fALFF, ReHo and DC differences between the two groups controlling for age, sex and education. Multiple comparisons were also corrected using a GRF theory, with a voxel *P*-value < 0.001, a cluster *P*-value < 0.05 and two-sided data.

#### Correlation analysis

Partial correlation analysis was used to analyse the correlation among the global NVC, the average ratio of significant clusters extracted by regional coupling and the cognitive scale scores in the presbycusis group after controlling for age, sex and education level, and the statistical significance was set at *P* < 0.05.

#### Reproducibility validation

Given that temporal and other grey matter volume (GMV) atrophy has been observed in individuals with presbycusis,^32,33^ global and regional NVC comparisons were repeated with the mean GMV of each subject, derived from segmentation of T1 structural images, as a covariate to regress the effect of GMV atrophy.

## Results

### Demographics and neurocognitive assessments

Finally, 36 patients with presbycusis and 36 HCs were included in this study. A summary of the demographic characteristics and neurocognitive status of all participants is provided in Table 1. There were no significant differences between the two groups in terms of age, sex or years of education. The PTA of the left and right ears and the average PTA of the presbycusis group were significantly greater than those of the HC group (*P* < 0.001, 1000-4000 Hz). However, in the neurocognitive evaluation, the presbycusis group exhibited worse CFT-delay, AVLT, AVLT-delay and DSST scores than the HC group (*P* = 0.010, 0.008, 0.000 and 0.004, respectively), and the time to finish the TMT-A and B was significantly longer than that of the HC group (*P* = 0.044 and 0.027, respectively). No significant differences were found for the other neurocognitive scale tests.

**Table 1:** Demographic and cognitive characteristics of presbycusis group and HCs

Items	Patients with presbycusis (*n* = 36)	Healthy controls (*n* = 36)	*P*-value
Age (years)	62.28 ± 7.39	59.58 ± 6.48	0.104
Gender (male/female)	21/15	16/20	0.346
Education (years)	8.64 ± 2.26	9.17 ± 2.65	0.366
GMV (cm^3^)	198.94 ± 27.12	214.40 ± 57.97	0.152
PTA of the right ear (dB HL)	39.79 ± 15.85	20.66 ± 3.52	0.000*
PTA of the left ear (dB HL)	40.97 ± 19.62	18.85 ± 2.86	0.000*
Mean PTA of both ears (dB HL)	40.38 ± 15.98	19.76 ± 2.07	0.000*
MoCA	25.11 ± 1.63	25.69 ± 1.74	0.147
DST	11.78 ± 2.09	12.31 ± 2.47	0.331
CFT	34.17 ± 2.62	34.39 ± 2.51	0.715
CFT-delay	7.42 ± 4.22	10.61 ± 5.86	0.010*
AVLT	21.50 ± 6.23	25.50 ± 6.11	0.008*
AVLT-delay	4.64 ± 2.39	6.92 ± 2.37	0.000*
DSST	34.28 ± 11.50	43.03 ± 13.41	0.004*
TMT-A	70.42 ± 28.00	58.64 ± 19.99	0.044*
TMT-B	184.36 ± 79.01	144.64 ± 69.64	0.027*
SAS	33.92 ± 5.46	33.69 ± 5.14	0.859
SDS	35.89 ± 6.49	34.33 ± 5.13	0.263

Data were represented as mean ± standard deviation except for gender.

GMV, grey matter volume; PTA, pure tone audiometry; MoCA, Montreal Cognitive Assessment; DST, digit span test; CFT, complex figure test; AVLT, auditory-verbal learning test; DSST, digit symbol substitution test; TMT-A, trail-making test A; TMT-B, trail-making test B; SAS, Self-rating Anxiety Scale; SDS, Self-rating Depression Scale.

*P < 0.05.

### Neurovascular coupling alterations at the whole-grey matter level in patients with presbycusis

**Figure 1:**
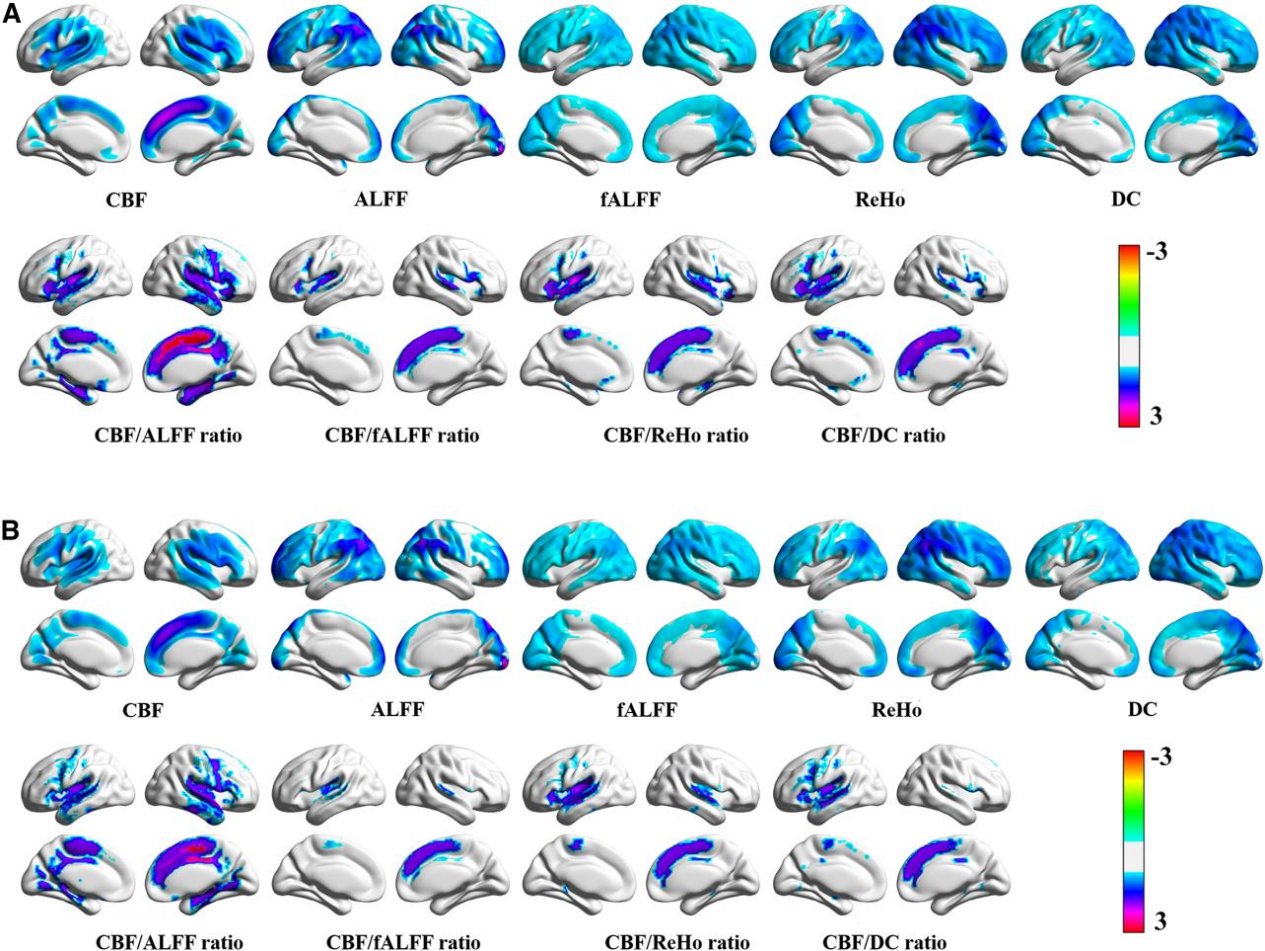
**Spatial distribution of averaged maps.** (**A**) Spatial distribution in patients with presbycusis. (**B**) Spatial distribution in HCs. CBF, cerebral blood flow; ALFF, amplitude of low-frequency fluctuation; fALFF, fractional ALFF; ReHo, regional homogeneity; DC, degree centrality.

**Figure 2:**
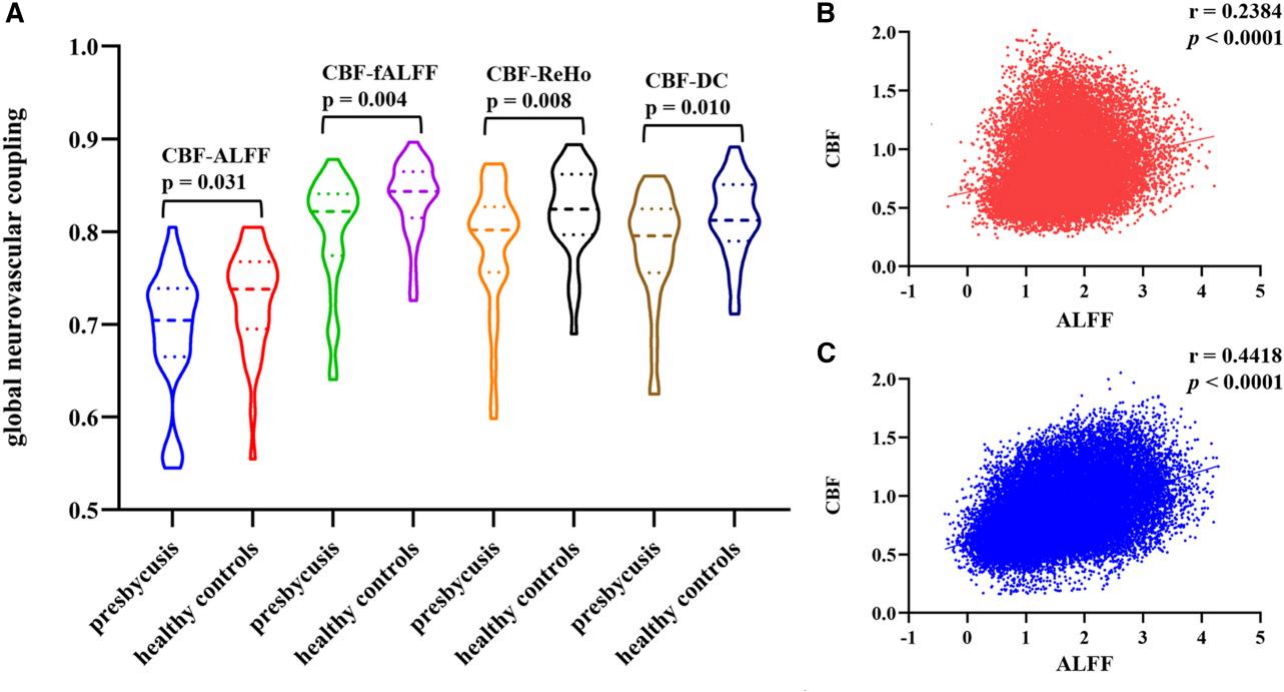
**Global neurovascular coupling and spatial correlation example graphs.** (**A**) At the group level, the mean global neurovascular coupling is significantly lower in patients with presbycusis than in HCs. For the comparison, an independent two-sample *t*-test was used and controlled for the effects of age, sex and education level. (**B, C**) Examples of the spatial correlation across voxels between CBF and ALFF in a patient and a control subject. CBF, cerebral blood flow; ALFF, amplitude of low-frequency fluctuation; fALFF, fractional ALFF; ReHo, regional homogeneity; DC, degree centrality.

The averaged global CBF, ALFF, fALFF, ReHo, binary DC, CBF/ALFF, CBF/fALFF, CBF/ReHo and CBF/DC ratio maps for both groups are presented in Fig. 1. Figure 2A shows the significant across-voxel correlations between CBF and metrics of neuronal activity, while Fig. 2B and C shows two representative correlation maps from one patient and one control. We investigated the manifestation of NVC at the whole grey matter level using four neurovascular metrics, namely CBF-ALFF, CBF-fALFF, CBF-ReHo and CBF-DC. Patients with presbycusis exhibited significantly reduced global coupling (*P* = 0.031, 0.004, 0.008 and 0.010, respectively) compared with the control group. In the regional coupling analysis, compared with HCs, patients with presbycusis exhibited a significant increase in the CBF/ALFF ratio in the right amygdala, an increase in the CBF/ReHo ratio in the left limbic lobe and an increase in the CBF/DC ratio in the left anterior cingulate gyrus, whereas reduced regional coupling was shown in the left precuneus in the four cohorts (Table 2).

**Table 2:** Brain areas with significant intergroup differences in CBF/ALFF, CBF/fALFF, CBF/ReHo and CBF/DC ratio

Group differences	Brain regions	Peak MNI coordinates (*x*, *y* and *z*)	Number of voxels (cluster size)	Peak intensity
CBF/ALFF ratio				
PC＞HC	Amygdala_R	24, 9, −15	172	4.6500
PC＜HC	Precuneus_L	−21, −90, 36	57	−5.4428
CBF/fALFF ratio				
PC＜HC	Precuneus_L	−18, −84, 30	90	−4.4831
CBF/ReHo ratio				
PC＞HC	Limbic Lobe_L	−6, 21, −9	70	4.7035
PC＜HC	Precuneus_L	−21, −84, 33	122	−5.224
CBF/DC ratio				
PC＞HC	Anterior Cingulate_L	−15, 24, −3	76	4.4142
PC＜HC	Precuneus_L	−21, −87, 36	103	−4.8433

Multiple comparisons were corrected according to Gaussian random field (GRF) theory, with voxel *P*-value < 0.001, cluster *P*-value < 0.05 and two-sided.

PC, presbycusis; HC, healthy controls; MNI, Montreal Neurological Institute; CBF, cerebral blood flow; ALFF, amplitude of low-frequency fluctuation; fALFF, fractional ALFF; ReHo, regional homogeneity; DC, degree centrality.

### Cerebral blood flow and resting-state functional MRI changes in patients with presbycusis

Despite showing similar spatial distribution, no significant difference was found in CBF or rs-fMRI measures alone between the two groups using the same correction method.

### Correlations between neurovascular coupling metrics and neurocognitive scores

**Figure 3:**
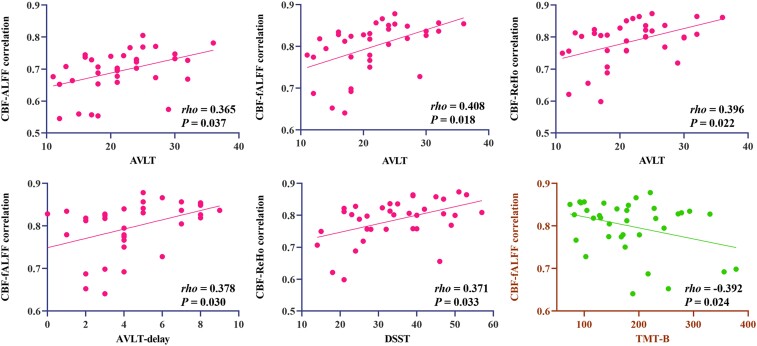
**Partial correlation between global NVC and cognitive measures in patients with presbycusis.** CBF, cerebral blood flow; ALFF, amplitude of low-frequency fluctuation; fALFF, fractional ALFF; ReHo, regional homogeneity; AVLT, auditory-verbal learning test; DSST, digit symbol substitution test; TMT-B, trail-making test B.

**Figure 4:**
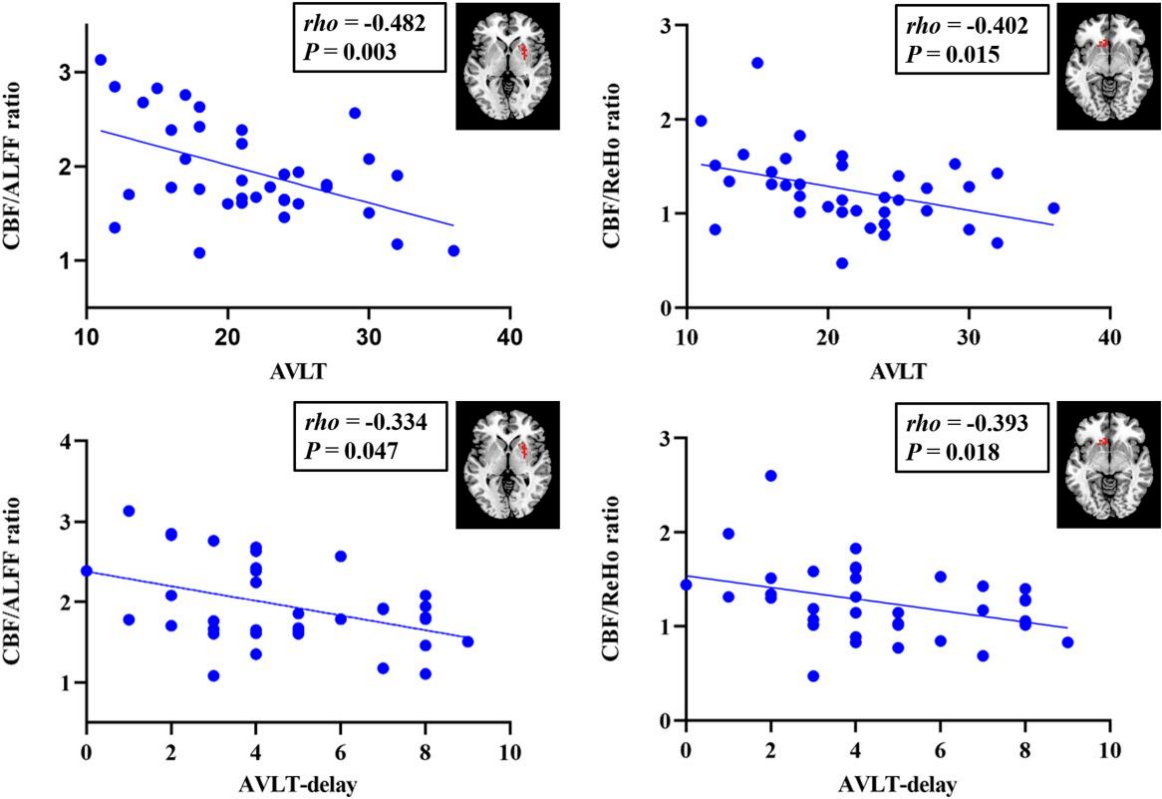
**Partial correlation between regional NVC and cognitive measures in patients with presbycusis.** The increased CBF/ALFF ratio in the right amygdala was negatively correlated with AVLT and AVLT-delay scores; the increased CBF/ReHo ratio in the left limbic lobe was negatively correlated with AVLT and AVLT-delay. CBF, cerebral blood flow; ALFF, amplitude of low-frequency fluctuation; fALFF, fractional ALFF; ReHo, regional homogeneity; AVLT, auditory-verbal learning test.

Partial correlation analysis was performed with significantly altered NVC metrics as target imaging biomarkers to examine their relationship with neurocognitive scale scores. According to the neuropsychological test results, both abnormally decreased NVC and increased NVC were related to cognitive decline in patients with presbycusis. Globally, the study showed significant positive correlations between CBF-ALFF, CBF-fALFF and CBF-ReHo coupling and AVLT scores (*rho* = 0.365, *P* = 0.037; *rho* = 0.408, *P* = 0.018; *rho* = 0.396, *P* = 0.022). Moreover, the CBF-fALFF coupling with AVLT-delay scores and the CBF-ReHo coupling with DSST scores were also positively correlated (*rho* = 0.378, *P* = 0.030; *rho* = 0.371, *P* = 0.033) (Fig. 3). In contrast, the CBF-fALFF coupling with TMT-B scores was negatively correlated (*rho* = −0.392, *P* = 0.024). Regionally, the abnormally increased CBF/ALFF ratio in the right amygdala was negatively correlated with the AVLT (*rho* = −0.482, *P* = 0.003) and AVLT-delay scores (*rho* = −0.334, *P* = 0.047); the abnormally increased CBF/ReHo ratio in the left limbic lobe was negatively correlated with the AVLT (*rho* = −0.402, *P* = 0.015) and AVLT-delay scores (*rho* = −0.393, *P* = 0.018) (Fig. 4).

### Validation results

Grey matter atrophy in patients with presbycusis suggested potential neuronal damage and loss of astrocytes, which may affect neurovascular coupling. In our study, although there was no significant difference in GMV between the presbycusis group and the HCs [(198.94 ± 27.12) cm^3^, (214.40 ± 57.97) cm^3^, *P* = 0.152], we repeated the intergroup comparison with GMV as an additional covariate of no interest. After controlling for GMV in each subject, there were still significant differences in global coupling between the two groups (CBF-fALFF: *P* = 0.010; CBF-ReHo: *P* = 0.016; CBF-DC: *P* = 0.022), except for CBF-ALFF coupling (*P* = 0.073). For regional coupling, after GMV correction, the spatial distribution of brain regions showing significant differences was consistent with the main results without GMV correction. The above results indicate that alterations in neurovascular coupling in patients with presbycusis are not related to changes in GMV.

## Discussion

NVU components are closely connected to form a complete anatomical and functional structural system that regulates CBF. Damage to any part will affect the overall operation. This study explored the alteration of NVC in patients with presbycusis, revealed the relationship between neuronal activity and cerebral blood perfusion and identified three main outcomes: the global coupling of whole GM in patients with presbycusis was lower than that in HCs; altered regional coupling mainly involved Papez circuit-related regions; NVC abnormalities are associated with cognitive impairment in patients with presbycusis. These findings help explain the neuropathology of cognitive impairment in patients with presbycusis.

The Papez circuit is a neural circuit that encompasses the hippocampus, mammillary body (MB), anterior thalamic nucleus (ATN) and cingulate gyrus. It is vital for emotional integration and memory tracking and tightly linked to learning and memory processes. Given that the thalamus and the descending auditory central pathway are closely linked to subcortical regions associated with emotional behaviour (e.g. amygdala, striatum and hypothalamus), reduced auditory input may compromise the functionality of critical components within the Papez circuit.^34^

### Dysfunction of the neurovascular coupling and its relevance to cognition

Significant across-voxel correlations between CBF and neuronal activity have been reported in the healthy brain, suggesting physiological neurovascular coupling.^15,35^ The NVC pattern represents the coordination between the brain’s oxygen requirements and blood supply, and abnormal coupling may reveal cerebral pathologies and neurological disorders.^30^ Similarly, compared with controls, patients with presbycusis exhibited significantly reduced global NVC, which is consistent with the findings of previous studies on most diseases,^35-37^ probably due to the following reasons: a decrease in the number of auditory neurons leads to decreased energy demand from neuronal activity, a decrease in neurovascular exchange and a decrease in coordination; additionally, hearing loss-induced damage to the blood–brain barrier and blood-labyrinth barrier affects cerebrovascular haemodynamic function, destroys cerebrovascular system integrity and leads to microvascular physiological homeostasis disturbances.^19^ This finding has also been interpreted as a consequence of the correlation between global coupling and cognition, a cognitive process that relies on the brain’s complex and fine neurovascular network, and damage to global coupling contributes to cognitive impairment in patients, primarily in the areas of attention switching and language learning.

Regional neurovascular coupling maintains a balanced state in the healthy brain, and either a decrease or an increase in this coupling implies a departure from equilibrium. In the present study, regional coupling dysfunction was revealed mainly by the fact that the CBF/ALFF, CBF/ReHo and CBF/DC ratios were significantly greater in the right amygdala, left limbic lobe and left anterior cingulate brain regions in the presbycusis group than in the HCs, while the CBF/ReHo ratio was significantly lower in the left precuneus. Interestingly, ALFF and fALFF reflect the intensity of spontaneous brain activity during resting states, while ReHo and DC measure local neuronal synchronization and network node centrality, respectively. In our study, no significant differences were observed in the simple comparison of CBF and rs-fMRI measurements, which is not entirely consistent with the reports in previous literature. This may be related to factors such as sample size, research methods and severity of the disease. Therefore, this study combined CBF with these indicators for coupling analysis. Neurovascular coupling is a dynamic process involving multiple interacting factors, which can understand brain function from the perspective of metabolism. We found that the regional NVC does provide more valuable information about neurovascular activity. Clinical correlation analyses also confirmed the devastating impact of changes in regional NVC, and NVC in altered brain regions was associated with impairment in the cognitive domain.

Activity in the amygdala, a central brain region involved in stress regulation and emotional responses, was strongly suppressed in the resting state. Under the chronic stress of increased hearing effort due to hearing loss, the amygdala fires and neurons activate accordingly, a process widely recognized as contributing to anxiety and depression^38^ and consistent with clinical expectations of poor emotional perception after hearing loss. Upregulation of coupling in this region means that the enhancement of amygdala neuronal activity led to an increase in CBF, which satisfies the need for higher energy and oxygen. CBF is one of the key factors in the NVC process. NVC describes the dynamic interaction between neuronal activity and vascular responses, where changes in CBF are an important manifestation of this interaction. When neuronal activity increases, CBF usually increases accordingly to meet the metabolic needs of neurons. Therefore, changes in NVC in the amygdala region may be directly influenced by variations in CBF. This in turn affects brain regions relevant to cognitive processing, leading to a cascading cognitive decline.

The limbic lobe contains structures such as the cingulate gyrus, hippocampus and parahippocampal gyrus. Dysfunctional connectivity of the auditory-limbic network has been found in patients with hearing loss,^39^ suggesting that the upregulation of coupling in this region originates from weakened neuronal activity. Fundamentally, impaired auditory cells may lose their role in information exchange between neurons and blood vessels, resulting in a synergistic alteration between functional and perfusion functions, which is reflected in the fact that sustained speech perception stress in hearing-impaired individuals leads to reduced adaptive neuronal activity in the limbic network involved in the emotional processing of sound. It can be concluded that changes in NVC are not always driven by a single factor.

Moreover, the anterior cingulate region is extensively connected to a neighbouring brain region, the prefrontal lobe, through bundles of white matter fibres. As neuronal activity declines, input stimuli to inferior brain regions decrease, resulting in a corresponding impairment of cognitive function mediated by the frontal lobe. The increased coupling in the anterior cingulate region reveals that the CBF in the corresponding brain region is decreased due to the decreased neural activity. The AVLT and AVLT-delay, used to validate memory encoding, storage and extraction capabilities, are useful scaling tools for the early identification of AD, and their relevance also confirms deficits in higher-order cognitive functions. Remarkably, we observed a downregulation of coupling in the precuneus, which is responsible for sophisticated cognition, such as episodic memory, self-related information processing and consciousness. The downregulation of these genes was speculated to be influenced by atrophy of the precuneus and the accumulation of tau protein,^32,40^ which in turn cause a reduction in CBF. Thus, it is essential to emphasize that NVC is a complex process influenced by multiple factors. While CBF and rs-fMRI are crucial indicators for assessing NVC, their variations do not always solely determine the state of NVC. Therefore, when interpreting changes in NVC, it is imperative to consider multiple factors.

In summary, the primary observation of Papez circuit involvement is reasonable, as patients with presbycusis are more often accompanied by mood disorders,^41,42^ cognitive deficits and difficulties in managing social relationships, which is consistent with previous neuroimaging findings.^43,44^

### Effect of grey matter on neurovascular coupling

The validation analysis showed that the change in NVC was essentially independent of the GMV, reflecting the relative stability of the present results. We described the neuroimaging properties of these cells from a global and regional perspective, providing detailed neurovascular information and promising insights for characterizing the neurovascular decoupling of the disease more integrally.

### Limitations

This study inevitably had several shortcomings. First, the findings were limited by a relatively small sample size and cross-sectional design, which affected the detailed description of NVC dysfunction in the population with presbycusis. Additional multicentre, large-sample test data will help improve the statistical power of the results. In addition, longitudinal studies should focus on the dynamics of cognitive function during long-term hearing loss to explore the effects of disease progression on the brain.^45^ Second, it must be acknowledged that the parameters of the images cannot be refined to the molecular level of fundamental research, while they can provide valuable information about metabolic efficiency, they are not directly equivalent to measures of neuronal activity, and more direct measures need to be explored in the future to deepen the understanding of brain function and metabolic mechanisms. Finally, we cannot completely rule out the potential effect of the degree of hearing loss on altered brain function;^46,47^ therefore, a subgroup analysis of patients may be needed.

## Conclusion

This study, through a comprehensive evaluation of BOLD and ASL data, revealed the disruption of neurovascular coupling in patients with presbycusis, explained the causes of aberrant neurocognitive performance and provided novel evidence for the neuropathological mechanism of cognitive impairment.

## Data Availability

All data will be available from the corresponding author on reasonable request and approval from the ethics committee. The data are not publicly available due to privacy or ethical restrictions.
